# From clinical reasoning to effective clinical decision making—new training methods

**DOI:** 10.3389/fpsyg.2015.00473

**Published:** 2015-04-21

**Authors:** Patricia P. Wadowski, Barbara Steinlechner, Arno Schiferer, Henriette Löffler-Stastka

**Affiliations:** ^1^Department of Clinical Pharmacology, Medical University of ViennaVienna, Austria; ^2^Division of Cardiothoracic and Vascular Anaesthesia and Intensive Care, Medical University of ViennaVienna, Austria; ^3^Department of Psychoanalysis and Psychotherapy, Medical University of ViennaVienna, Austria

**Keywords:** eLearning, teaching case, clinical reasoning, acute thrombocytopenia, residency

## The clinical problem

Identification of causes and immediate adjustment to treatment of acute thrombocytopenia occurring in patients in the intensive care unit is required to avoid imminent complications. Hence it is important to train awareness and clinical decision making of students in the medical curriculum. Therefore, real-life cases were transferred into an interactive eLearning platform comprising the steps of patient assessment and therapeutic decisions.

Heparin-induced platelet count decrease is an immune-mediated prothrombotic disorder, resulting from an adverse drug reaction (Kelton and Warkentin, 2008). After cardiac surgery antibodies against circulating heparin—platelet factor (PF) four complexes develop in up to 50%. Patients experience a risk of 1–5% to acquire clinical symptoms of heparin-induced thrombocytopenia (HIT) (Warkentin et al., 2000; Linkins et al., 2012). Due to complications, mortality rates are high and amount to 5–10% (Kelton and Warkentin, 2008; Kelton et al., 2013).

As clinical teaching case a 59-year-old male patient is presented, who was admitted to the intensive care unit (ICU) of the General Hospital of Vienna on extracorporeal life support (ECMO). The man underwent bypass surgery six days ago in a peripheral hospital and is concomitantly suffering from an active infection. On the fourth day at ICU a platelet count decrease has been noticed.

## Identification of the problem

Students are now challenged to identify possible differential diagnoses and further to apply the 4Ts-scoring system. The 4Ts comprise the components Thrombocytopenia, Timing, Thrombosis, and other causes of thrombocyte decrease (Lo et al., 2006; Cuker et al., 2012). Clinical manifestation of HIT is usually characterized by a platelet count decline of 50% or more in 24 h, and moderate to severe thrombocytopenia, which is evoked by disseminated intravascular coagulation accompanied with micro- or macro- embolic events (Warkentin, 2015). Thrombocytopenia after cardiac surgery is expected to occur on the first postoperative days (nadir platelet counts up to 4 days after surgery) (Warkentin, 2015). In contrast, the onset of heparin—associated thrombocytopenia typically emerges 5–10 days after drug administration.

Two patterns are described: a biphasic curve of platelet count course, where a second thrombocyte fall begins about 4 days after surgery or a persistence of thrombocytopenia longer than 4 days post-operatively (Lillo-Le Louet et al., 2004). Furthermore, HIT is associated with thrombotic events in up to 75% of patients, where severe and often atypical thrombosis (e.g., bilateral deep vein thrombosis, pulmonary embolism, arterial thrombosis followed by ischemic limb necrosis) is observed (Warkentin et al., 1995; Warkentin, 2015).

## Disease pathophysiology

The basis of clinical reasoning is a profound knowledge of disease pathomechanisms and evoked complications. It is important to realize that HIT is a *prothrombotic* disorder, mediated by IgG binding to conformationally exposed epitopes on PF4, a chemokine secreted by activated platelets. The circulating PF4-heparin- antibody complexes cross-link Fc-receptors expressed on the cell surface of mainly platelets and monocytes. Microparticles containing procoagulant proteins are released and promote further activation of the coagulation cascade (Kelton et al., 2013). The pathophysiology of HIT is demonstrated using flowcharts of the described processes.

## Clinical decision making

Clinical decision making should proceed based on the results of the 4T-score—along the clinical problem based on associative and procedural learning processes: This scoring system has a high negative predictive value, hence it is recommended to continue heparin treatment in patients scored below four points (Alatri et al., 2012; Cuker et al., 2012). In cases of high clinical HIT- suspicion (scoring above four points), alternative anticoagulation treatment has to be immediately initiated. With the help of multiple-choice questions students are instructed to discontinue the heparin infusion instantly and change to the direct thrombin inhibitors argatroban (as first-line choice) or bivalirudin in patients with hepatic impairment (Coutre et al., 2015). Other treatment options are the factor Xa inhibitors danaparoid or fondaparinux (Linkins et al., 2012; Coutre et al., 2015). Furthermore, students' attention is drawn to identify all possible sources of heparin (coating of the suction and irrigation systems/catheters). For this form of question-driven learning multiple-choice questions are used, including feedback for each question in order to provide stepwise help.

## Laboratory analyses

After clinical assessment, blood samples should be sent to the medical laboratory. It is important to fill in the form correctly and to communicate the 4T score and a brief patient history as well as the clinical course to the Laboratory Physicians. At the Department of Blood Group Serology of the Medical University of Vienna the analyses are adapted to the points achieved in the 4Ts score; quicktests (rapid lateral flow tests) as screening for patients scored below four points and ELISA assays are performed.

## Interdisciplinary questions

Patients suspected of heparin-induced thrombocytopenia need continuous monitoring with regard to platelet count and new thrombosis (Alatri et al., 2012). Neurological status assessment shall be performed at least once daily and herein we also recommend the use of the Glasgow Coma Score (GCS) and the Richmond Agitation Sedation Scale (RASS) (Teasdale and Jennett, 1974; Reade and Finfer, 2014). Indices of thrombosis often require consultation and close cooperation with internal specialists/ neurologists and vascular surgeons for thrombectomy performance, if needed.

## The clinical course of the patient

The anticoagulation of the patient was immediately changed to a direct thrombin inhibitor and patient hematological and neurological status monitored closely. Platelet count courses (in relation to fibrinogen) are shown to students with interactive evaluation possibility. For training purposes, the case includes a patient suffering from instantly occurring HIT-complications, i.e., intracerebral thromboembolism, where students are confronted with the symptoms and possible diagnostic methods.

## Concept and methodological approach

Clinical reasoning is considered to consist of intuitive and analytical components (Croskerry, 2009; Kassirer, 2010). Research on mental processes suggests that disease patterns are stored in “frames,” “clinical scenarios,” “semantic networks/qualifiers,” or “illness scripts.” Repeated presentation and exercising of clinical cases is known to be crucial for an efficient learning process (Norman, 2005; Kassirer, 2010).

Implementation of the interactive case-based teaching method into the Medical Curriculum in Vienna was initiated in 2014. For teaching purposes, real-life clinical patient cases are transferred from the general hospital information management into an online platform provided by the Medical University of Vienna. Data anonymisation is performed automatically through the transmission system. Teachers and students provide knowledge to add background information, program relevant questions as well as a feedback system to give direction to the reasoning process.

Results associated with the evaluation of these newly adjusted and improving curriculum-elements (Turk et al., 2015) after introducing case-based exercises and case-based questioning showed a positive steering effect on the learning process and fostered the acquisition of associative and procedural knowledge (Seitz and Löffler-Stastka, 2015). The main effect on the learning process was given through the implementation of affectively involving case-based exercises discussed in seminars fostering clinical reasoning processes (Parth et al., 2014) and preparing students for patient-centered thinking in the case of following bedside teaching methods.

## Message

To summarize, students should practice everyday patient evaluation and learn the value of interdisciplinary team communication as well as the application of the 4Ts score and crucial disease pathophysiology.

In the future, the concept of interactive case-based learning methods should be developed further and established as a training program for residents. Case-based question-driven learning provides a solid basis for associative and procedural learning that is necessary in clinical reasoning processes. In order to provide a diversity of case-based learning materials several other domains establish their learning cases in eLearning/blended learning scenarios. The basic didactic principle and the institutional framework were installed project-related (Turk et al., 2015) and received merits within the award-system for communication 2015.

### Conflict of interest statement

The authors declare that the research was conducted in the absence of any commercial or financial relationships that could be construed as a potential conflict of interest.

## Abbreviations

AKIM: general hospital information management (a clinical database)
ECMO: extracorporeal life support
ELISA: enzyme-linked immunosorbent assay
GCS: Glasgow Coma Score
HIT: heparin-induced thrombocytopenia
ICU: intensive care unit
IgG: immunoglobulin G
PF 4: platelet factor 4
RASS: Richmond Agitation Sedation Scale.
